# Validation of fibroblast activation protein and α‐smooth muscle actin as prognostic biomarkers in prostate cancer through AI‐assisted image analysis of dual‐marker IHC

**DOI:** 10.1002/2056-4538.70068

**Published:** 2025-12-17

**Authors:** Jenni Säilä, Timo‐Pekka Lehto, Antti Rannikko, Olli Kallioniemi, Tuomas Mirtti, Teijo Pellinen

**Affiliations:** ^1^ Institute for Molecular Medicine Finland (FIMM), Helsinki Institute of Life Science (HiLIFE) University of Helsinki Helsinki Finland; ^2^ Research Program in Systems Oncology (ONCOSYS) and iCAN – Digital Precision Cancer Medicine Flagship University of Helsinki Helsinki Finland; ^3^ Department of Urology University of Helsinki and Helsinki University Hospital Helsinki Finland; ^4^ Science for Life Laboratory, Department of Oncology and Pathology Karolinska Institute Solna Sweden; ^5^ Department of Pathology, Diagnostic Center Helsinki University Hospital Helsinki Finland; ^6^ Finnish Cancer Institute Helsinki Finland

**Keywords:** prostate cancer, tumour microenvironment, fibroblast activation protein (FAP), alpha‐smooth muscle actin (αSMA), prognostic biomarkers, artificial intelligence, deep‐learning, digital pathology

## Abstract

Prostate cancer (PCa) lacks reliable and accurate tissue‐based biomarkers to support prognostic stratification and clinical treatment decisions. Current diagnostic assessment, including Gleason grading, has limitations such as interobserver variability and insufficient granularity for disease aggressiveness. Fibroblast activation protein (FAP) and α‐smooth muscle actin (αSMA) have emerged as putative stromal biomarkers, but their prognostic value in localised PCa has not been validated at scale. In this study, we developed a novel artificial intelligence (AI)‐augmented image analysis pipeline tailored for dual‐marker immunohistochemistry of FAP and αSMA, enabling automated, tissue compartment‐specific quantification of biomarker expression. This deep learning model was trained and validated using digitised high‐resolution whole‐slide images of tissue microarrays from three prostatectomy cohorts, comprising 4,097 cores from 835 patients with comprehensive clinical follow‐up data. The AI pipeline demonstrated high accuracy in detecting epithelial, stromal, and immune compartments, as well as in quantifying FAP and αSMA signals. We validated stromal FAP as a robust prognostic marker consistently associated with adverse clinical outcomes, including earlier biochemical recurrence, metastasis, and cancer‐specific death. Epithelial FAP and stromal αSMA showed additional prognostic associations in selected analyses, particularly in MRI‐visible tumours. Our findings reinforce the biological and clinical relevance of stromal FAP in the prostate tumour microenvironment. By enabling standardised and scalable biomarker quantification, our newly developed AI‐assisted workflow advances the clinical utility of FAP and αSMA and demonstrates the power of integrating digital pathology with biomarker quantification. This study represents a critical step toward implementing stromal biomarkers in routine PCa diagnostics and underscores the potential of AI‐enhanced histopathology in advancing precision oncology.

## Introduction

Prostate cancer (PCa) is the second most frequently diagnosed cancer among men worldwide [1]. Despite advances in detection and treatment, clinical management of many individual PCa patients remains challenging due to the lack of robust biological prognostic markers. This limitation contributes to both overtreatment and undertreatment, as disease aggressiveness is often difficult to assess [2, 3, 4, 5]. Currently, the Gleason grading system remains the cornerstone of risk stratification in PCa [6]. However, Gleason grading is inherently subjective, and most tumours are classified within intermediate Grade Groups (GG 2–3), where prognostic uncertainty is greatest [3, 7]. Consequently, there is a critical need for reproducible, tissue‐based biomarkers that can improve risk stratification and guide more personalised treatment strategies.

Fibroblast activation protein (FAP) and alpha smooth muscle actin (αSMA) have emerged as promising biomarkers for aggressive and deadly PCa [8, 9]. FAP is a transmembrane serine protease expressed by reactive stromal fibroblasts and plays a key role in extracellular matrix remodelling, facilitating tumour invasion and metastasis through degradation of structural proteins and modulation of the tumour microenvironment [10, 11, 12, 13, 14]. Its overexpression across multiple cancer types – particularly within the stroma – has established FAP as a hallmark of cancer‐associated fibroblasts (CAFs) [12, 15]. In addition, FAP (historically described as the membrane‐bound protease seprase) expression has been reported in tumour cells of several malignancies, including pancreatic, gastric, colorectal, breast, and melanoma cancers [16, 17, 18]. Elevated FAP levels are consistently associated with aggressive disease and poor clinical outcomes [19]. In PCa, stromal FAP is enriched in metastatic castration‐resistant tumours and correlates with disease progression [9, 20, 21].

αSMA (ACTA2), a cytoskeletal protein, is a marker of smooth muscle cells and myofibroblasts and is commonly used to identify activated stromal fibroblasts [22, 23, 24]. In the tumour microenvironment, αSMA‐positive myofibroblasts contribute to tumour progression *via* secretion of growth factors and extracellular matrix remodelling [25, 26]. Interestingly, unlike in other solid tumours, αSMA expression in PCa decreases during disease progression, likely reflecting the loss of native smooth muscle in the prostate stroma rather than a classic CAF expansion [8, 9, 27, 28, 29]. Together, the differential expression patterns of FAP and αSMA suggest a unique stromal remodelling process in PCa, with potential implications for their utility as prognostic biomarkers.

The emergence of deep learning (DL) and neural networks has enabled new approaches in digital pathology, including the automated analysis of immunohistochemistry (IHC) for biomarker quantification [30]. These AI‐based tools offer improved reproducibility and sensitivity over manual assessment by accurately segmenting tissue compartments, identifying cellular features, and quantifying biomarker expression in a standardised manner [31].

Building upon our previous findings [8, 9], the current study aims to validate FAP and αSMA as prognostic tissue biomarkers in localised PCa using a novel convolutional neural network (CNN)‐based AI model for dual‐marker IHC analysis. The model was trained to perform tissue compartment‐specific detection of FAP and αSMA, as well as leukocyte infiltration, in digitised high‐resolution images from three prostate cancer tissue microarray (TMA) cohorts. This AI‐augmented image analysis pipeline enables reproducible, scalable biomarker quantification and advances the clinical translation of FAP and αSMA for PCa prognostication.

## Materials and methods

### Patient cohorts and TMA construction

#### MRI cohort

This cohort included 387 patients diagnosed using multiparametric MRI and treated with robot‐assisted laparoscopic prostatectomy (RALP) at Helsinki University Hospital (HUS) between 2014 and 2015. TMA design from RALP specimens was previously described by Eineluoto *et al* [32]. Patient‐matched TMA samples were collected from MRI‐visible (three cores), MRI‐invisible (two cores), and tumour‐adjacent ‘normal’ (one core, hereafter named benign) regions. After quality control of excluding bad quality cores (e.g., folded, unfocused, unrepresentative), the cohort consisted of 348 patients with comprehensive clinical data (Table 1).

**Table 1:** Clinicopathological characteristics of the study cohorts

Gleason	MRI	HKI1	HKI2		
	GG	GS	GG	Metastatic (GG)	Non‐metastatic (GG)
GS ≤6/GG1, *n* (%)	14 (4%)	85 (27%)			
GS7/GG2, *n* (%)	139 (40%)	186 (58%)	66 (39%)	25 (30%)	41 (48%)
GS7/GG3, *n* (%)	154 (44%)		70 (42%)	41 (50%)	29 (34%)
GS8/GG4, *n* (%)	10 (3%)	37 (12%)	32 (19%)	16 (20%)	16 (19%)
GS9/GG5, *n* (%)	31 (9%)	10 (3%)			
Undefined		1 (0%)			
Total, *n*	348	319	168	82	86
Age					
Median (IQR)	68.5 (8.6)	64 (8.0)	62.7 (9)	62.2 (9.7)	63.5 (8.0)
PSA (ng/ml)					
Median (IQR)	8.6 (6.7)		9.2 (6.5)		
Follow‐up time (years)					
Median (IQR)	3.4 (1.0)	16.5 (5.5)	10.4 (5.6)		
pTNM					
pT2	211 (61%)	189 (59%)	59 (35%)	26 (32%)	33 (38%)
≥pT3	137 (39%)	129 (41%)	109 (65%)	56 (68%)	53 (62%)

GS, Gleason score; GG, Grade Group; IQR, interquartile range; pTNM, pathological tumour‐node‐metastasis.

#### HKI1 cohort

This cohort comprised PCa patients from a continuous population‐based series of radical prostatectomies at HUS between 1983 and 1998. FFPE samples were archived at the Department of Pathology. TMA generation was described by Sahu *et al* [33], including two cores from the dominant cancer area, one from a secondary cancer area, and one from benign tissue. Following quality control, 319 patients with complete clinical data were included (see Table 1).

#### HKI2 cohort

This cohort included 168 patients with Grade Group (GG) 2–4 localised PCa treated with open or robot‐assisted prostatectomy at HUS between 1992 and 2015. Patients were stratified into metastatic (*n* = 82) and non‐metastatic (*n* = 86) groups based on follow‐up events (see Table 1 for clinical data). TMA design was previously reported by Lehto *et al* [34].

### Immunohistochemistry and slide scanning

FAP and αSMA dual‐colour IHC was performed on formalin‐fixed paraffin‐embedded (FFPE) TMA sections from all three cohorts (835 patients, 4,097 TMA cores) at the Digital Microscopy and Molecular Pathology Unit, Institute for Molecular Medicine Finland (FIMM, Helsinki, Finland). The protocol employed a dual‐chromogen IHC assay for detecting FAP and αSMA protein expression on 3.5‐μm FFPE sections. After antigen retrieval and blocking steps, tissue sections were incubated with a mixture of primary antibodies against FAP (clone EPR20021, Abcam) and αSMA (clone 1A4, Agilent Dako). Chromogenic detection was carried out using DAB for FAP and Liquid Permanent Red for αSMA, followed by haematoxylin counterstaining.

Slides were digitised using a Pannoramic P250 Flash III whole‐slide scanner (3DHistech, Budapest, Hungary) with a ×20 objective (NA 0.8). The scanned images were uploaded to the Aiforia® cloud‐based platform (Aiforia Technologies, Helsinki, Finland) for neural network training and image analysis. Validation of the FAP antibody was previously performed using CRISPR‐Cas9 knockout in the WPMY‐1 prostate stromal cell line [35].

Detailed reagent information, incubation times, and procedural steps are provided in Supplementary Materials and Methods.

### Image analysis

Image analysis was conducted using Aiforia Create, a cloud‐based deep‐learning platform (Aiforia Technologies). We developed a two‐part CNN algorithm employing pixel‐level multi‐class semantic segmentation. Training of the model was based on manual pixel‐level annotations. The first neural network was trained to segment epithelial tissue, stroma, and leukocyte clusters, while the second network detected chromogenic signals for FAP and αSMA. The final model nested the signal detection within tissue compartments for compartment‐specific quantification. As epithelial αSMA was not observed, only stromal αSMA and both epithelial and stromal FAP were analysed. For the final model, two CNNs were integrated: the ‘tissue neural net’ segmented epithelium, stroma, leukocyte clusters, and background, whereas ‘the signal neural net’ detected DAB (FAP) and red chromogen (αSMA) signals.

Training data were selected to capture diverse tissue morphologies across three cohorts. The Tissue neural net was trained through 440 manual annotations representing different tissue areas, including epithelium, stroma, lymphocyte clusters, and image backgrounds, to identify the actual sample area. The teaching set included a total of 14 slides from Cohort 1 and 2 slides each from Cohort 2 and 3. The signal net was trained with 54 manual annotation regions representing FAP and αSMA positive signal in seven slides from Cohort 1.

Model performance was evaluated against independent manual annotations on a set of 26 regions and a separate test set of 16 TMA cores. Precision (P), sensitivity/recall (S/R), and F1 were computed pixel‐wise (area‐based) for tissue classes and chromogen‐positive signal, using the standard definition [F1 = 2·P·R/(P + R)]. Final quantification results were expressed in square millimetres and normalised to stromal or epithelial compartment areas.

### Statistical analysis

This study followed the Reporting Recommendations for Tumour Marker Prognostic Studies (REMARK) guidelines [36]. All statistical analyses were performed using RStudio (R version 4.1.3; Posit Software, Boston, MA, USA) and Microsoft Excel.

Data normality was assessed using the Kolmogorov–Smirnov test. The Wilcoxon rank‐sum test (Mann–Whitney *U* test, unpaired) was used to evaluate differences between groups. Spearman's rank correlation was applied to assess associations between continuous variables. The chi‐square test was used for categorical variable comparisons.

Survival analyses included Kaplan–Meier estimation for biochemical recurrence (BCR)‐free, metastasis‐free, and cancer‐specific survival. The Cox proportional hazards regression model was used to evaluate associations between covariates and survival outcomes. Statistical significance of individual covariates in prognostic analysis was assessed using the likelihood ratio test, Wald test, and log‐rank test. The proportional hazards assumption in multivariable Cox models was evaluated using the Schoenfeld residuals test. A two‐sided *p* value of <0.05 was considered statistically significant throughout.

Biomarker expression was calculated as the ratio of FAP‐ or αSMA‐positive area to total stromal or epithelial area, as appropriate. For each patient, the maximum value from index lesion TMA cores was used for tumour samples, while benign tissue values were derived from a single core per patient.

For tissue architecture comparisons in Cohort 1 (MRI cohort), the relative areas of stroma, epithelium, and leukocyte clusters were quantified and normalised to the total tissue area of each TMA spot. These comparisons were used to assess differences between MRI‐visible, MRI‐invisible, and benign regions.

### Ethics approval

Research approvals for the use of archived samples and clinical data were granted by the Finnish National Supervisory Authority for Welfare and Health (Valvira; Dnro V/38176/2018 and FIMEA/2022/006397). The study was conducted without individual informed consent, in accordance with Finnish and European Union legislation on non‐interventional medical research. Ethical approval was provided by the Institutional Ethics Committee of the Hospital District of Helsinki and Uusimaa (HUS), under approvals HUS/1439/2018, HUS/419/2018, HUS/850/2017, HUS/155/2021, and HUS/124/2023.

## Results

### AI algorithm performance and tissue compartment quantification

**Figure 1:**
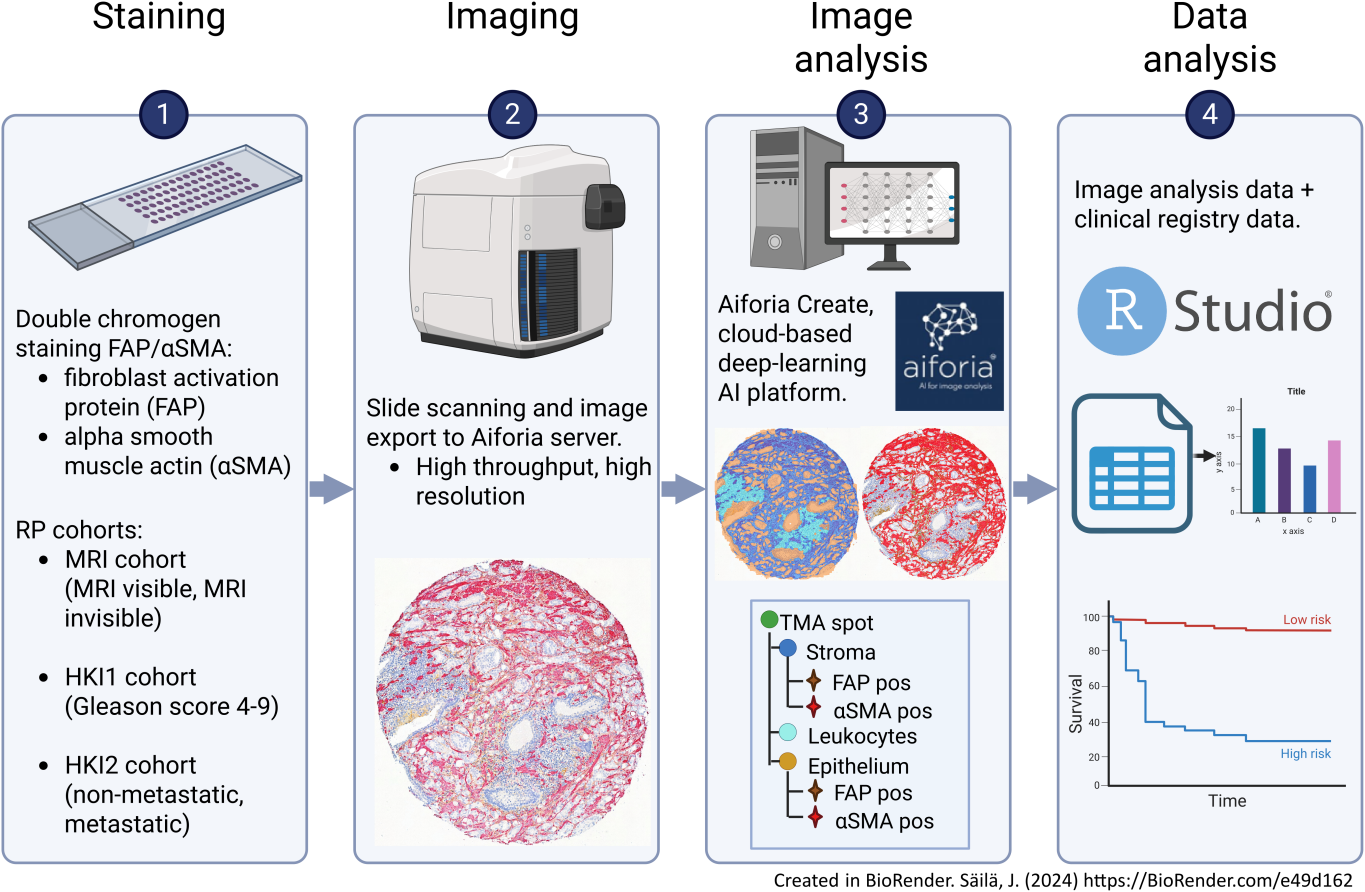
Overview of the study workflow. Schematic illustration of the project pipeline, including dual‐marker immunohistochemical staining for fibroblast activation protein (FAP) and α‐smooth muscle actin (αSMA) on prostate cancer tissue microarrays (TMAs), slide digitisation, and AI‐based tissue compartment–specific image analysis using the Aiforia platform. Quantitative image data were integrated with clinical and pathological variables across three patient cohorts: MRI‐visible/invisible tumours (Cohort 1), Gleason Grade–based tumours (HKI1), and metastasis outcome–based tumours (HKI2). Figure created with Biorender (www.biorender.com, license agreement number NX2931RJRF)

We developed an AI‐based image analysis pipeline for automated quantification of FAP and αSMA expression in prostate cancer TMAs stained using a dual‐chromogen IHC protocol (Figure 1). The Aiforia DL model demonstrated high precision and sensitivity in segmenting tissue compartments and detecting biomarker‐positive areas. For the tissue detection CNN model precision, sensitivity and F1 score (harmonic mean of precision and sensitivity) were 98.5%, 98.6%, and 98.55%, respectively. For the biomarker signal detection model, precision, sensitivity, and F1 score were 89.9%, 96.3%, and 92.94%. These values reflect pixel‐wise comparisons of predicted masks to independent manual annotations in the validation regions and test TMA cores.

**Figure 2:**
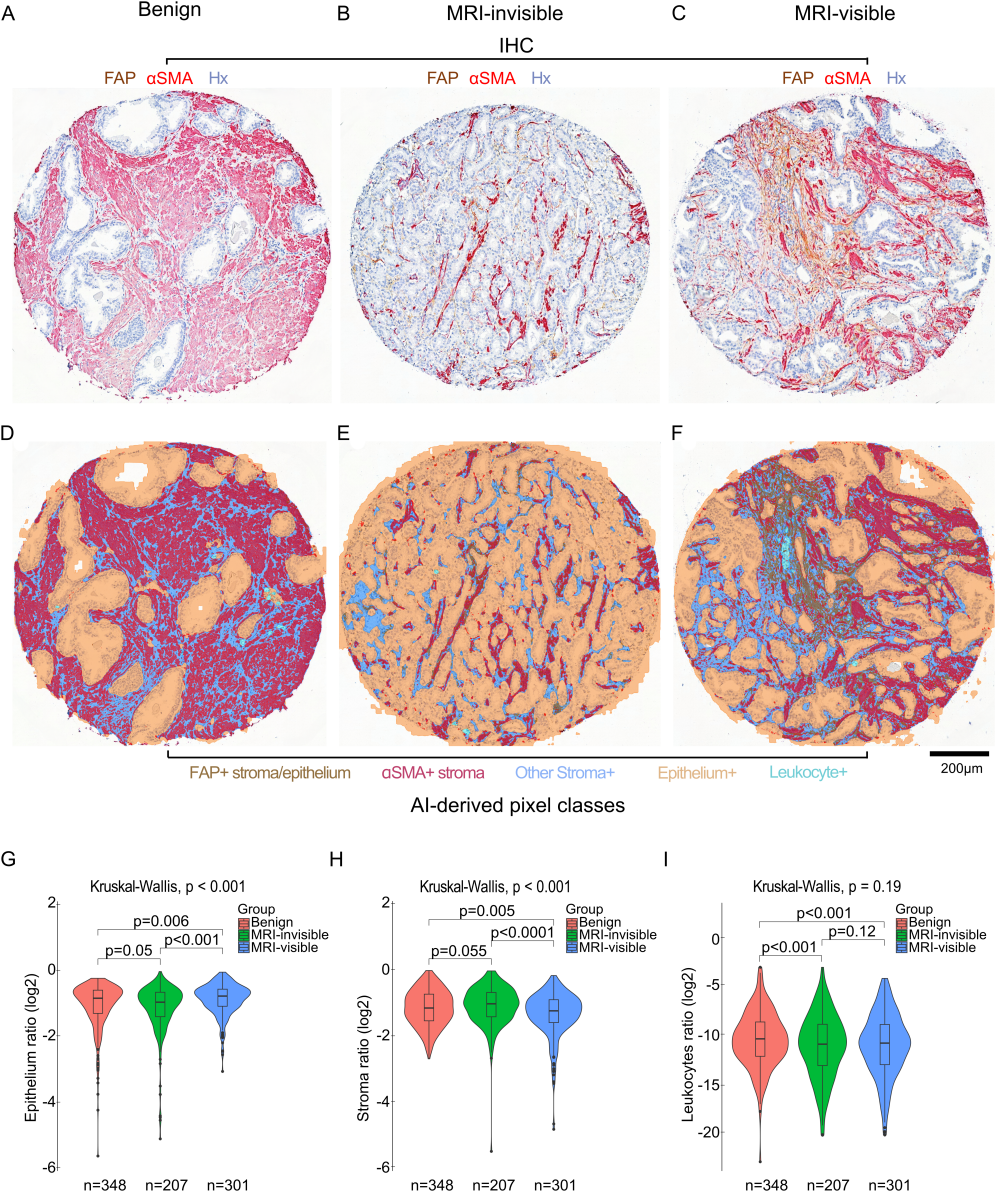
AI‐augmented tissue compartment quantification. (A–C) Representative TMA cores from (A) benign, (B) MRI‐invisible, and (C) MRI‐visible tumours stained with FAP (brown), αSMA (red) and Haematoxylin (Hx, blue). (D–F) Tissue segmentation and biomarker detection in corresponding TMA cores, showing coloured mask images for classified pixels of stromal/epithelial FAP (brown), stromal αSMA (red), leukocyte clusters (cyan), other stroma (light blue), and epithelium (FAP negative). (G–I) Quantification of (G) epithelial, (H) stromal, and (I) leukocyte areas among benign (*n* = 348), MRI‐invisible (*n* = 207), and MRI‐visible (*n* = 301) cores. Statistical comparisons were performed using the Kruskal–Wallis test or Mann–Whitney *U* test, as appropriate. Violin plots show data distribution; box plots show medians and IQRs (interquartile ranges).

In Cohort 1 (MRI cohort), the model accurately identified epithelial, stromal, and leukocyte regions (Figure 2A–F). Quantification of relative epithelial area (normalised to total tissue area) revealed highest values in MRI‐visible tumour cores (mean = 0.60, SD = 0.16), followed by MRI‐invisible (mean = 0.50, SD = 0.17), and benign regions (mean = 0.50, SD = 0.18) (Kruskal–Wallis test, *p* < 0.001; Figure 2G, H). Leukocyte cluster density was significantly lower in benign samples (mean = 0.0018, SD = 0.009) compared to MRI‐visible (mean = 0.0024, SD = 0.006) and MRI‐invisible (mean = 0.0025, SD = 0.010) tumour regions (Mann–Whitney *U* test, *p* < 0.0001 for both comparisons; Figure 2I).

### Biomarker expression patterns and association with recurrence

**Figure 3:**
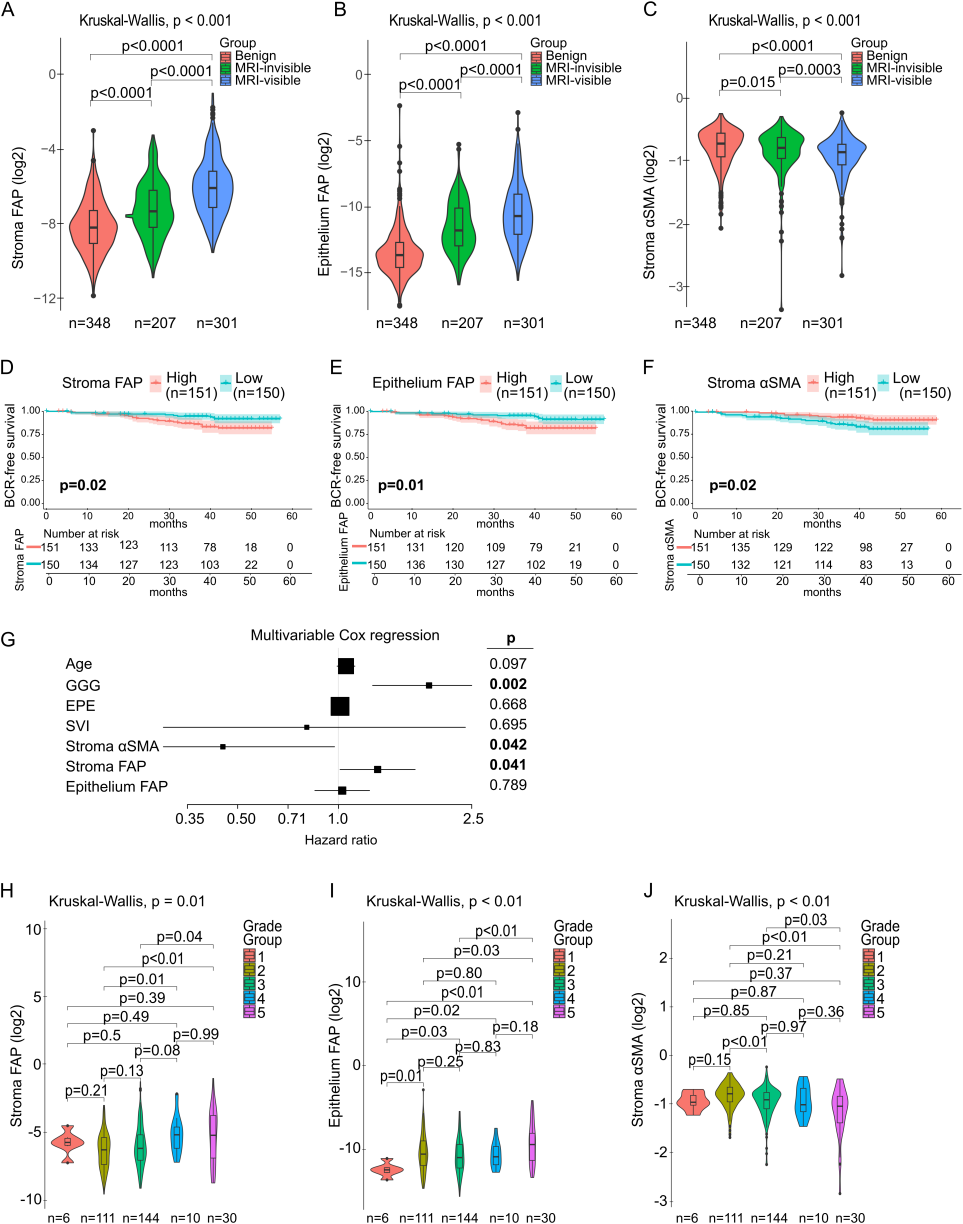
Biomarker expression patterns and association with recurrence. (A–C) Log2‐transformed FAP and αSMA positive areas in benign (*n* = 348), MRI‐invisible (*n* = 207), and MRI‐visible (*n* = 301) cores in Cohort 1. Kruskal–Wallis test for global and Wilcoxon rank‐sum test for two‐group comparisons. Box plots illustrate the median and IQR. (D–F) Kaplan–Meier curves for BCR‐free survival based on stromal FAP, epithelial FAP, and stromal αSMA in MRI‐visible cores, dichotomised at the median (high *n* = 151, low *n* = 150), cohort 1. Log‐rank test. (G) Multivariable Cox regression of continuous biomarker values adjusted for age, GGG, EPE, and SVI in MRI‐visible tumours (*n* = 301), Cohort 1. Wald test. (H–J) FAP and αSMA distributions across Gleason Grade Groups (GG1 *n* = 6, GG2 *n* = 111, GG3 *n* = 144, GG4 *n* = 10, GG5 *n* = 30) in Cohort 1. Kruskal–Wallis test for global and Wilcoxon rank‐sum test for two‐group comparisons. Box plots illustrate the median and IQR. BCR, biochemical recurrence; GG, Grade Group; EPE, extraprostatic extension; SVI, seminal vesicle invasion; IQR, interquartile range.

Analysis of Cohort 1 revealed that stromal FAP expression (normalised to total stroma area) was highest in MRI‐visible tumours [mean = 0.029, interquartile range (IQR) = 0.020], followed by MRI‐invisible tumours (mean = 0.011, IQR = 0.010), and benign regions (mean = 0.006, IQR = 0.010) (Kruskal–Wallis test, *p* < 0.001; Figure 3A). Epithelial FAP expression (relative to epithelium) was also significantly higher in MRI‐visible tumours (mean = 0.0027, IQR = 0.0016) compared to MRI‐invisible (mean = 0.0009, IQR = 0.0008) and benign regions (mean = 0.0008, IQR = 0.0001) (Kruskal–Wallis test, *p* < 0.001; Figure 3B). Stromal αSMA expression decreased from benign (mean = 0.59, IQR = 0.15) to MRI‐invisible (mean = 0.57, IQR = 0.13) and MRI‐visible (mean = 0.54, IQR = 0.12) tumours (Kruskal–Wallis test, *p* < 0.001; Figure 3C). Histograms of FAP and αSMA distributions are shown in supplementary material, Figure S1A–C. Stromal and epithelial FAP levels were positively correlated (Spearman's *ρ* = 0.53–0.60, *p* < 0.001; see supplementary material, Figure S1D–F for full correlation plots of stromal and epithelial FAP).

Kaplan–Meier survival analysis in MRI‐visible tumours showed that high stromal and epithelial FAP were associated with shorter BCR‐free survival (log‐rank test, *p* = 0.02 and *p* = 0.01, respectively), whereas high stromal αSMA was associated with longer BCR‐free survival (*p* = 0.02; Figure 3D–F). Cox regression using continuous values confirmed these associations: stromal FAP [hazard ratio (HR) = 1.55, 95% CI: 1.26–1.91, *p* < 0.001], epithelial FAP (HR = 1.22, 95% CI: 1.06–1.41, *p* = 0.005), and stromal αSMA (HR = 0.22, 95% CI: 0.12–0.41, *p* < 0.001; Table 2). In MRI‐invisible tumours, stromal FAP remained prognostic for BCR‐free survival (HR = 1.36, 95% CI: 1.02–1.83, *p* = 0.038), while epithelial FAP and stromal αSMA were not significantly associated (Table 2 and supplementary material, Figure S2). In benign TMA regions, none of the biomarkers reached statistical significance, though stromal FAP showed a trend (HR = 1.24, 95% CI: 0.98–1.57, *p* = 0.077; Table 2).

**Table 2:** Univariable Cox regression analysis of FAP and αSMA expression for predicting BCR in the MRI cohort

Group	Variable	HR	95% CI	*p*
MRI‐visible (*n* = 301)	Epi FAP	1.22	1.06–1.41	**0.005**
	Stroma FAP	1.55	1.26–1.91	**<0.001**
	Stroma αSMA	0.22	0.12–0.41	**<0.001**
MRI‐invisible (*n* = 207)	Epi FAP	1.11	0.89–1.38	0.362
	Stroma FAP	1.36	1.02–1.83	**0.038**
	Stroma αSMA	0.57	0.24–1.32	0.190
Benign (*n* = 344)	Epi FAP	1.05	0.89–1.25	0.563
	Stroma FAP	1.24	0.98–1.57	0.077
	Stroma αSMA	0.51	0.19–1.40	0.190
Clinical variables (*n* = 348)	Age	1.08	1.02–1.14	**0.013**
	GG	2.22	1.65–2.98	**<0.001**
	Prostate weight	0.99	0.97–1.02	0.569
	PSA pre‐prostatectomy	1.03	1.00–1.06	0.078
	EPE	1.08	1.04–1.11	**<0.001**
	Perineural invasion	2.27	0.54–9.45	0.262
	SVI	3.25	1.56–6.78	**0.002**

Bold font indicates statistically significant *p* values (*p* < 0.05). FAP and αSMA expression were analysed as continuous variables using univariable Cox proportional hazards regression.

BCR, biochemical recurrence; CI, confidence interval; EPE, extra‐prostatic extension; Epi, epithelium; GG, Grade Group; HR, hazard ratio; MRI, magnetic resonance imaging; SVI, seminal vesicle invasion.

Multivariable Cox regression in MRI‐visible tumours, adjusted for age, GG, extra‐prostatic extension, and seminal vesicle invasion, confirmed stromal FAP (*p* = 0.041) and stromal αSMA (*p* = 0.042) as independent prognostic factors (Figure 3G). Stratification by GG in MRI‐visible tumours showed that both stromal and epithelial FAP were positively associated with increasing grade (Kruskal–Wallis test, *p* < 0.01; Figure 3H, I), while stromal αSMA was enriched in lower‐grade tumours (*p* < 0.01; Figure 3J).

### External validation of prognostic utility in independent cohorts

**Figure 4:**
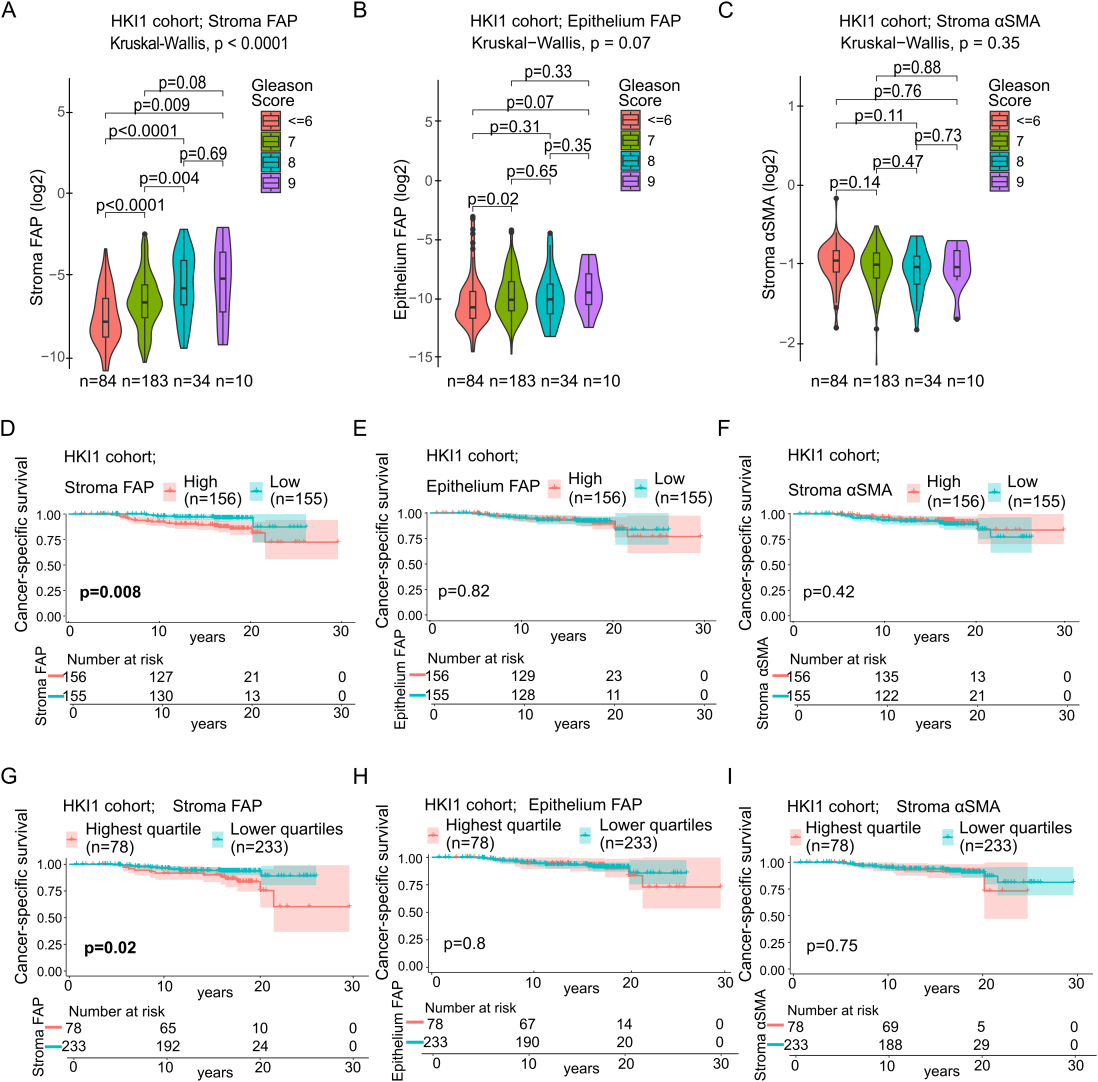
External validation of FAP as a prognostic tissue biomarker for cancer‐specific survival. (A–C) Log2‐transformed biomarker distributions by Gleason Score in Cohort 2 (HKI1) (GS ≤6 *n* = 84, GS7 *n* = 183, GS8 *n* = 34, GS9 *n* = 10). Kruskal–Wallis test for global and Wilcoxon rank‐sum test for two‐group comparisons. Box plots illustrate the median and IQR. (D–F) Kaplan–Meier curves for cancer‐specific survival based on median dichotomisation of stromal FAP, epithelial FAP, and stromal αSMA (high *n* = 156, low *n* = 155) in Cohort 2 (HKI1). Log‐rank test. (G–I) Kaplan–Meier curves for cancer‐specific survival based on top quartile versus remainder dichotomisation of stromal FAP, epithelial FAP, and stromal αSMA (high *n* = 78, low *n* = 233) in Cohort 2. Log‐rank test. GS, Gleason Score.

In Cohort 2 (HKI1), stromal FAP was positively associated with Gleason Score (Kruskal–Wallis test, *p* < 0.001; Figure 4A). Epithelial FAP and stromal αSMA did not show statistically significant associations (*p* = 0.07 and *p* = 0.35, respectively; Figure 4B, C). Stromal FAP predicted cancer‐specific survival using both median (log‐rank test, *p* = 0.008) and upper quartile cut‐offs (*p* = 0.02; Figure 4D, E). Epithelial FAP and stromal αSMA did not show significant associations with cancer‐specific survival (Figure 4F–I).

**Figure 5:**
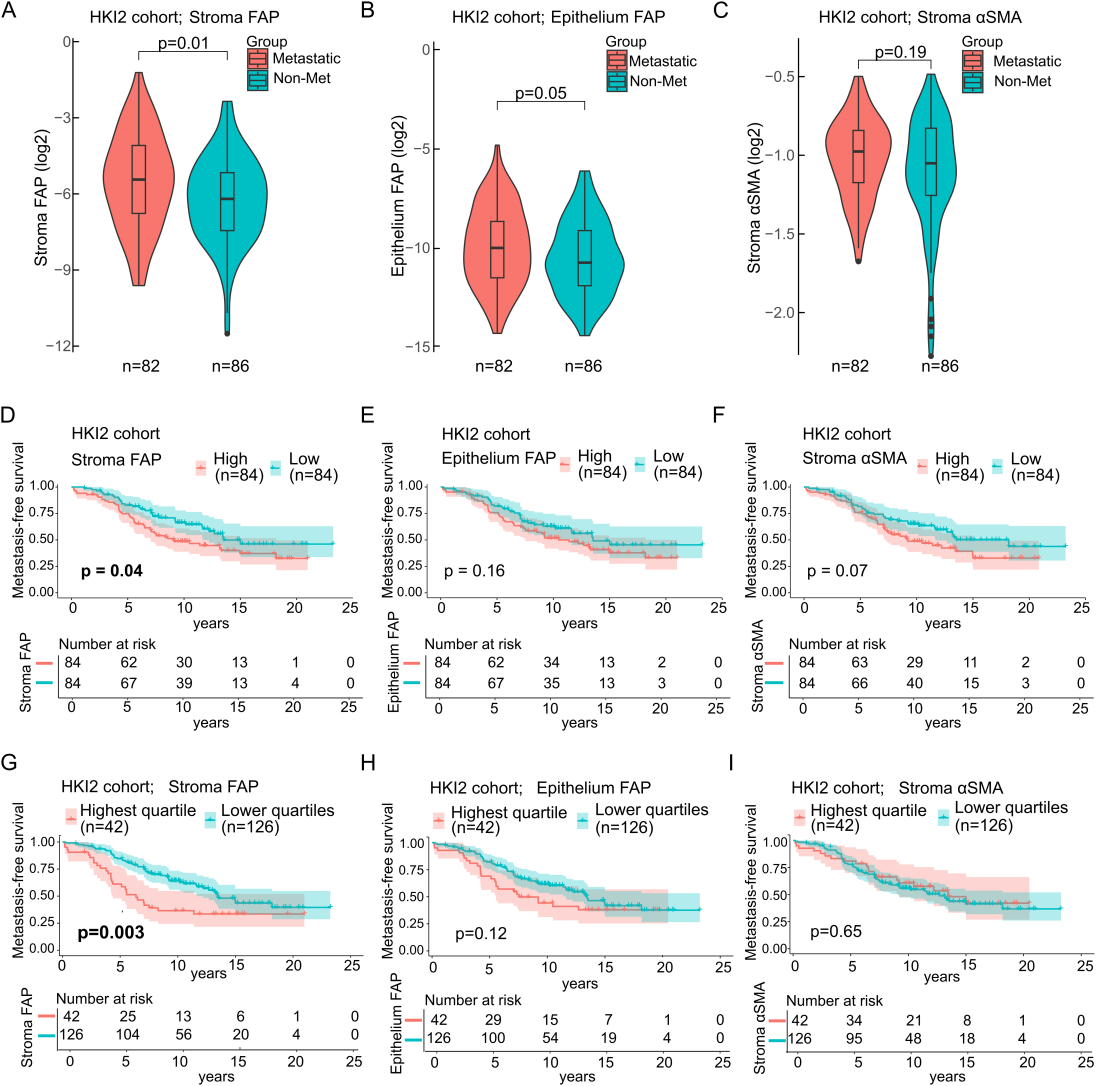
External validation of FAP as a prognostic tissue biomarker for metastasis‐free survival. (A–C) Log2‐transformed biomarker distributions in metastatic (*n* = 82) versus non‐metastatic (*n* = 86) patients in Cohort 3 (HKI2). Wilcoxon rank‐sum test. (D–F) Kaplan–Meier curves for metastasis‐free survival by median dichotomisation of stromal FAP, epithelial FAP, and stromal αSMA (high *n* = 84, low *n* = 84) in Cohort 3. Log‐rank test. (G–I) Kaplan–Meier curves for metastasis‐free survival by top quartile versus remainder dichotomisation of stromal FAP, epithelial FAP, and stromal αSMA (high *n* = 42, low *n* = 126) in Cohort 3. Log‐rank test.

In Cohort 3 (HKI2), stromal FAP expression was higher in metastatic versus non‐metastatic cancers (Mann–Whitney *U* test, *p* = 0.01; Figure 5A), but epithelial FAP and stromal αSMA were not statistically different between metastatic and non‐metastatic cancers (*p* = 0.05 and *p* = 0.19, respectively; Figure 5B, C). Stromal FAP was associated with shorter metastasis‐free survival (log‐rank test, median cut‐off: *p* = 0.04; upper quartile: *p* = 0.003; Figure 5D, E). Epithelial FAP and stromal αSMA did not predict metastasis‐free survival (Figure 5F–I).

Across all three cohorts, stromal FAP consistently demonstrated independent prognostic value for disease progression, while epithelial FAP and stromal αSMA showed more limited or cohort‐specific associations.

## Discussion

### Main findings

Our study validates the significance of FAP as a prognostic tissue biomarker in prostate cancer (PCa) using an AI‐based image analysis model. We established a novel dual‐marker IHC, enabling AI‐based automated detection of FAP and αSMA positivity in a tissue compartment‐specific manner (epithelium, stroma). Furthermore, the model also enabled automated detection of leukocyte clusters. We analysed tumours and matched tumour‐adjacent benign areas in 835 PCa patients within three different TMA cohorts. Our findings demonstrate that, in addition to stromal FAP, epithelial FAP expression is also significantly associated with MRI visibility and BCR. Finally, we showed that high stromal FAP is associated with shortened cancer‐specific and metastasis‐free survival, making it a superior IHC marker over epithelial FAP and stromal αSMA.

### Validation of earlier observations

Our findings validate and extend our earlier results [9], where we demonstrated using the same dual‐marker FAP/αSMA IHC assay and Random Forest‐based machine learning model that stromal FAP is associated with both BCR‐free survival and cancer‐specific survival. The validation of these findings with additional cohorts further supports the utility of FAP as a prognostic biomarker and highlights the robustness of our newly developed DL‐based analysis in providing reliable and reproducible results.

Our CNNs achieved F1 = 98.6% (tissue/region segmentation) and F1 = 92.9% (biomarker‐signal detection), which are comparable to other IHC DL systems. For example, a recent Ki‐67 study [37] reported a pixel‐wise tissue classifier F1 = 0.858 on a held‐out test set, while other IHC scoring systems report F1 = 0.82–0.91 depending on the task and ground truth [38, 39, 40].

As FAP is considered a marker for CAFs [10, 12, 13, 14] and may also be expressed in tumour cells across various tissue types [16, 17, 18], we created a novel AI‐based image analysis model to recognise FAP separately in both tissue compartments (epithelium/stroma). Unlike in our earlier work, with the DL‐based compartment‐specific analysis we observed an association of poor BCR‐free survival with stromal FAP not only in MRI‐visible tumour regions but also in MRI‐invisible tumours. Additionally, there was a trend towards significance when FAP was analysed in tumour‐adjacent benign areas, where elevated expression of FAP has been shown in proliferative inflammatory atrophy (PIA) and reactive fibrotic stroma [41]. Our results suggest that the compartment‐specific DL‐based tool provides more detailed and statistically significant insights compared to the earlier analysis tool.

Our results showing that high FAP expression correlates with poor prognosis and aggressive disease in PCa, are consistent with other previous studies. High FAP expression in metastatic castration‐resistant prostate cancer and its association with poor prognosis has been reported by Vlachostergios *et al* [20] and Hintz *et al* [42]. Similar trends have been observed in other cancers such as non‐small cell lung cancer (NSCLC) [43] and colorectal cancer [44], further supporting the role of FAP as a marker of aggressive disease. Additionally, we found that stromal FAP is positively associated with Gleason Grade, indicating that higher FAP levels correlate with more clinically significant tumours in prostate cancer. This reinforces the utility of FAP as a biomarker for identifying high‐risk PCa patients. In castration‐resistant prostate cancer, where FAP IHC often remains positive, including in prostate‐specific membrane antigen (PSMA)‐negative tumours, fibroblast activation protein inhibitor positron emission tomography (FAPI‐PET) can detect such lesions, highlighting FAP's complementary role to PSMA imaging in late‐stage disease [42, 45, 46].

However, some studies have reported contradictory results regarding FAP's role in other cancers. For example, Kilvaer *et al* found high FAP to be an indicator of positive outcome in some subtypes of NSCLC squamous cell carcinoma hypothesising different histologic subgroups of CAFs being present [15]. Also, in pancreatic ductal adenocarcinoma (PDAC), high FAP correlated with prolonged survival in some PDAC patients [47]. In invasive ductal carcinoma of the breast, a similar positive association between high stromal FAP expression and improved patient survival was observed [48].

Interestingly, across all three prostate cancer cohorts, we observed a consistent association between leukocyte cluster density and epithelial FAP expression, while stromal FAP showed weaker or cohort‐dependent associations. This suggests that FAP may be upregulated in response to local immune signals, possibly as part of a cytokine‐driven ‘field effect’ influencing both stromal and epithelial compartments. The strong correlation between epithelial and stromal FAP expression further supports this notion.

Previous work has linked stromal FAP to immune cell infiltration, including CD8^+^ T cells and CD163^+^ macrophages, in MRI‐visible prostate tumours [9], lung adenocarcinoma [49], and in clear cell renal cell carcinoma [35]. Recent studies further show that inflammatory cytokines – such as IL‐1β, TGF‐β, CCL2, and FGFBP1 – can induce FAP expression *via* NF‐κB, STAT3, and FGFR1–ERK signalling pathways [50, 51, 52, 53]. In prostate cancer, FAP^+^ stromal programmes co‐occur with immunosuppressive myeloid states (e.g., CD163^+^/SPP1^+^ macrophages) [54] and FAP itself can also promote macrophage activation and tissue remodelling [55]. These data support a bidirectional crosstalk between FAP‐expressing cells and the immune compartment, with myeloid cells, particularly macrophages, emerging as key mediators of this interaction.

### Implications for clinical practice

Currently, there are no IHC biomarkers universally adopted in clinical practice for prostate cancer treatment stratification. The integration of AI in histopathology promises to revolutionise tissue biomarker analysis by offering improved accuracy, reproducibility, and efficiency [56, 57]. AI‐based methods are already being integrated into clinical workflows for various cancers. For example, Aiforia has developed CE‐IVD marked Ki‐67, ER, and PR AI models for breast cancer, as well as a PD‐L1 recognition algorithm for lung cancer, that have been approved for medical use [58, 59]. Notably, DL methods are characterised by their adaptability, allowing for efficient retraining to accommodate diverse sample sets. The potential for AI to produce reproducible results is critical for its broader application in clinical settings [60]. The implementation of AI‐based automated systems could streamline the diagnostic process, reduce inter‐observer variability, and enable more personalised treatment strategies [61, 62].

### Challenges and future directions

Despite the promising results, our study acknowledges limitations and potential biases due to the use of the same patient cohorts for training, validation, and analysis. In addition, the experiments were done in the same laboratory by the same personnel, minimising differences in staining and image quality. External validation with independent cohorts is necessary to confirm the generalisability of our AI‐based algorithm. Although TMA samples are an effective method for introducing diverse tissue morphologies in a compact and cost‐effective manner [63], future research should focus on applying these methods to biopsies and whole slide sections to ensure robustness and applicability in diverse clinical scenarios.

## Conclusions

By integrating automated dual‐marker staining, digital high‐resolution imaging, and AI‐based tissue compartment‐specific image analysis, our pipeline, when integrated into clinical workflows, could make personalised prostate cancer medicine more effective and accessible. Our work not only reaffirms the relevance of FAP in PCa progression and prognosis but also illustrates the transformative potential of AI in refining diagnostic and therapeutic strategies in oncology.

## Author contributions statement

TP conceived the study. JS performed algorithm training, and formal analysis with the assistance of T‐PL. TP and JS interpreted the data and wrote the manuscript with input from TM. TM, T‐PL and AR provided clinical and pathological data and contributed to the study design. TP and TM supervised the study. OK provided administrative support and resources. All authors were involved in writing the paper and had final approval of the submitted and published versions.

## Supporting information


Supplementary materials and methods.
**Figure S1.** FAP and αSMA expression distributions and correlations
**Figure S2.** Biochemical recurrence‐free survival

## Data Availability

Data generated in this study will be deposited in Zenodo and made publicly available upon acceptance of the manuscript.
