# Supplementary material for: Validation of fibroblast activation protein and α‐smooth muscle actin as prognostic biomarkers in prostate cancer through AI‐assisted image analysis of dual‐marker IHC
Source: J Pathol Clin Res. 2025 Dec 17;12(1):e70068. doi: 10.1002/2056-4538.70068 (PMC12712236; doi:10.1002/2056-4538.70068)
Supplement: Supplementary file 1 — Supplementary materials and methods. Figure S1. FAP and αSMA expression distributions and correlations Figure S2. Biochemical recurrence‐free survival [file CJP2-12-e70068-s001.pdf]

Validation of fibroblast activation protein and α-smooth muscle actin as prognostic biomarkers in prostate cancer through AI-assisted image analysis of dual-marker IHC

J Säilä *et al*. *J Pathol Clin Res* https://doi.org/10.1002/2056-4538.70068

Supplementary materials and methods

Supplementary Figures S1 and S2

Supplementary materials and methods

Immunohistochemistry protocol for dual FAP and αSMA staining

Tissue sectioning

Formalin-fixed, paraffin-embedded (FFPE) tissue microarray (TMA) blocks were sectioned at 3.5 μm thickness using a microtome and mounted on Superfrost™ Ultra Plus adhesive slides (Fisher Scientific, Waltham, MA, USA).

Deparaffinisation and rehydration

Slides were deparaffinised in xylene (2 × 10 min) and rehydrated through a graded ethanol series (100%, 95%, 70%) followed by distilled water.

Blocking

Endogenous peroxidase activity was blocked with 0.3% hydrogen peroxide for 15 min at room temperature. Non-specific binding was blocked using 5% normal goat serum (NGS; Gibco, Thermo Fisher Scientific, cat. #16210064) for 15 min.

Primary antibody incubation

Slides were incubated for 90 min at room temperature with the following primary antibody mix:

- Anti-FAP rabbit monoclonal antibody (clone EPR20021, Abcam, 1:300 dilution)
- Anti-αSMA mouse monoclonal antibody (clone 1A4, Agilent Dako, 1:500 dilution)

Secondary antibody incubation and detection

Slides were incubated for 30 min with:

- HRP-conjugated anti-rabbit IgG (DPVR55HRP)
- AP-conjugated anti-mouse IgG (DPVM55AP)

  (Both from Immunologic, a WellMed Company)

Chromogenic development

- FAP: visualised using DAB (Bright Vision, BS04-110) for 8 min
- αSMA: visualised using Liquid Permanent Red (Agilent Dako, K0640) for 5 min

Counterstaining and mounting

Slides were counterstained with Mayer’s haematoxylin (1:10 dilution; Agilent Dako, S3309), rinsed in water, air-dried, and mounted using Pertex medium (Histolab Products).

Slide scanning

Slides were scanned at 20× magnification (NA 0.8) using a Pannoramic P250 Flash III scanner (3DHistech, Budapest, Hungary) and uploaded to the Aiforia® platform (Aiforia Technologies, Helsinki, Finland).

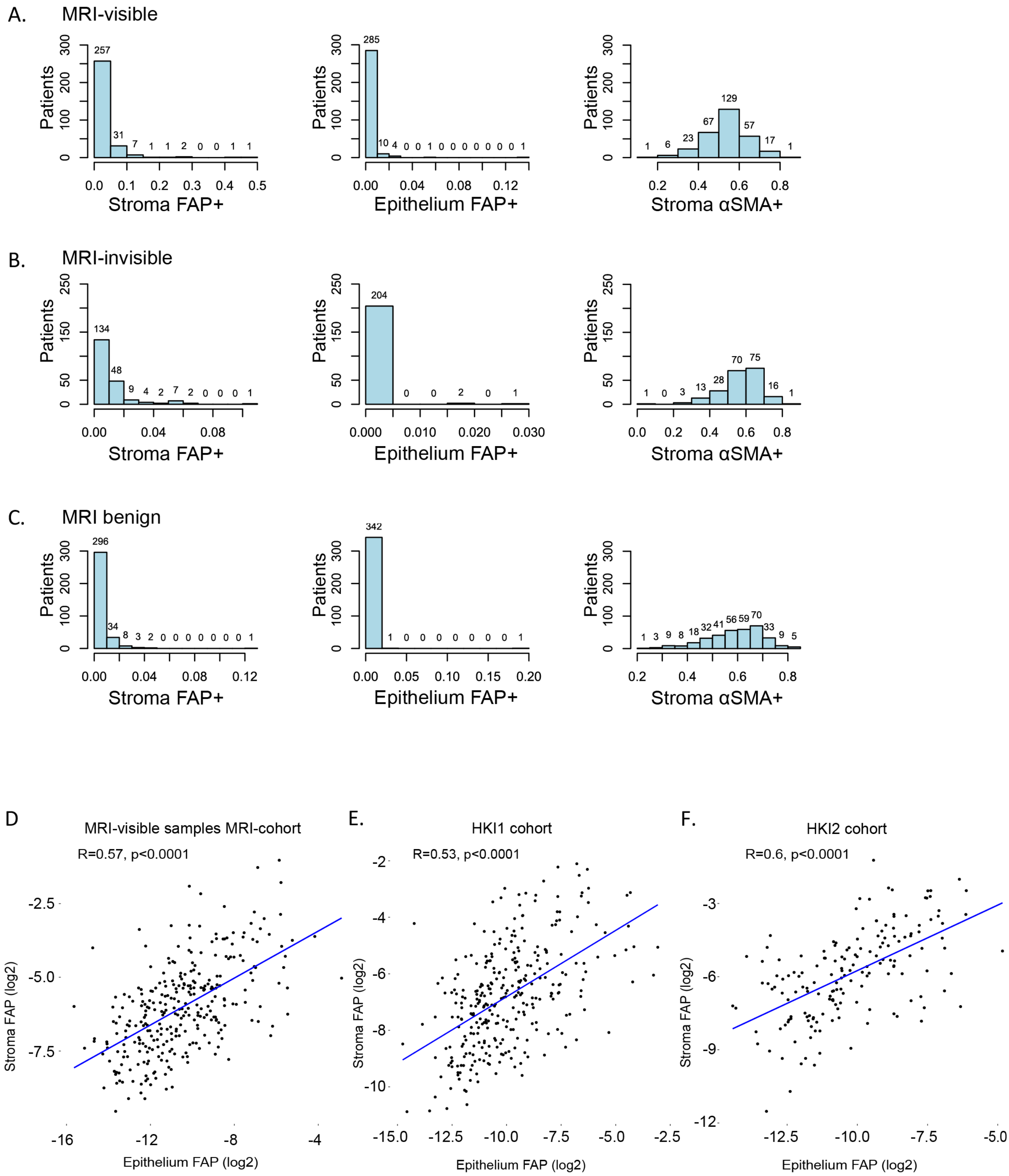

Figure S1. FAP and αSMA expression distributions and correlations of stromal and epithelial FAP.

(A–C) Histograms showing expression distributions in MRI-visible (*n* = 301), MRI-invisible (*n* = 207), and benign (*n* = 344) samples from Cohort 1.

(D–F) Spearman correlations of stromal versus epithelial FAP in MRI-visible (Cohort 1, *n* = 301), HKI1 (*n* = 319), and HKI2 (*n* = 168). Correlation coefficients and *p* values shown.

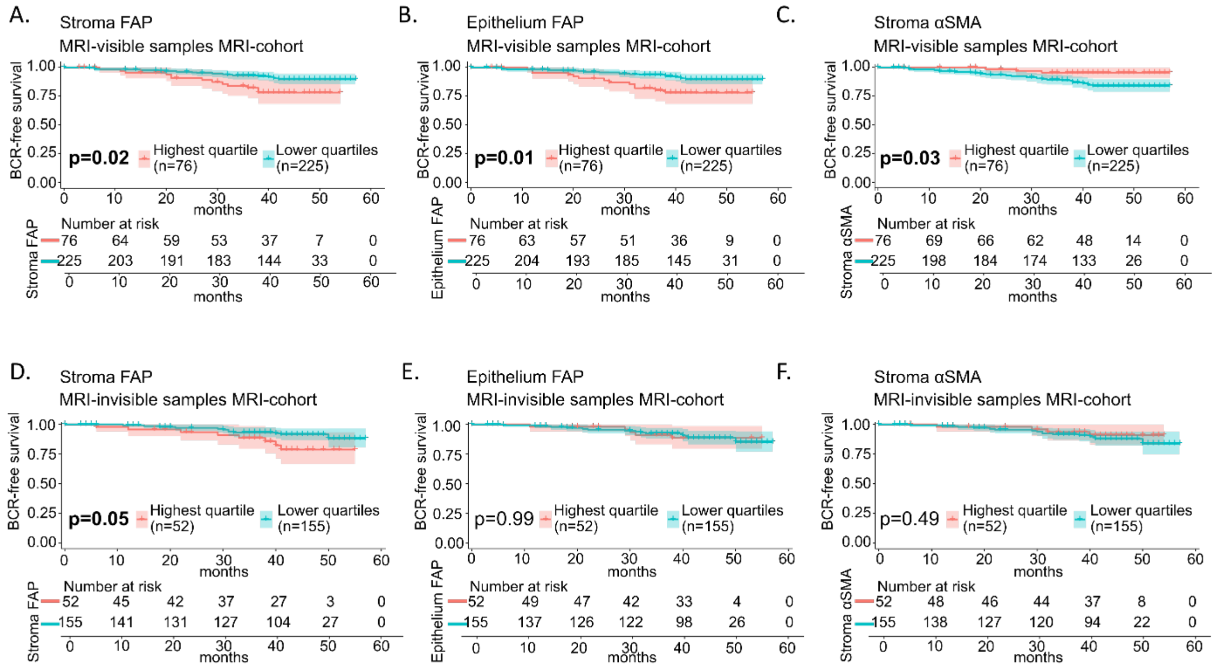

Figure S2. Biochemical recurrence-free survival in MRI cohort.

(A–C) Kaplan–Meier curves for MRI-visible tumours (highest quartile n = 76, lower quartiles n = 225).

(D–F) Kaplan–Meier curves for MRI-invisible tumours (highest quartile n = 52, lower quartiles n = 155). p values from log-rank test.
